# Response of water and photosynthetic physiological characteristics to leaf humidification in *Calligonum ebinuricum*

**DOI:** 10.1371/journal.pone.0285130

**Published:** 2023-05-04

**Authors:** Huimin Wang, Zhoukang Li, Suwan Ji, Guanghui Lv

**Affiliations:** College of Ecology and the Environmental, Xinjiang University, Urumqi, China; Universidade Federal de Alfenas, BRAZIL

## Abstract

Foliar water uptake (FWU) has increasingly been regarded as a common approach for plants to obtain water under water-limited conditions. At present, the research on FWU has mostly focused on short-term experiments; the long-term FWU plant response remains unclear; Methods: Through a field in-situ humidification control experiment, the leaves of *Calligonum ebinuricum* N. A. Ivanova ex Soskov were humidified, and the changes of leaf water potential, gas exchange parameters and fluorescence physiological parameters of plants after long-term and short-term FWU were discussed; The main results were as follows: (1) After short-term humidification, the water potential of *Calligonum ebinuricum* decreased, the non-photochemical quenching (*NPQ*) increased, and the plant produced photoinhibition phenomenon, indicating that short-term FWU could not alleviate drought stress. (2) After long-term humidification, the leaf water potential, chlorophyll fluorescence parameter and net photosynthetic rate (*Pn*) increased significantly. That is to say, after long-term FWU, the improvement of plant water status promoted the occurrence of light reaction and carbon reaction, and then increased the net photosynthetic rate (*Pn*); Therefore, long-term FWU is of great significance to alleviate drought stress and promote *Calligonum ebinuricum* growth. This study will be helpful to deepen our understanding of the drought-tolerant survival mechanism of plants in arid areas.

## Introduction

Stone [1] first reported that plants absorb water through leaves in the 1950s (foliar water uptake: FWU). However, most early scholars believed that plant growth and development depended mainly on root water uptake, and that FWU did not have physiological effects [2]. With the research and development of physiological ecology, researchers found that when the leaves are in a high humidity environment or there is water on the surface, leaves can absorb water through pores [3], cuticles [4], trichomes [5], drains and suction holes [6]. There are three possible fates for water absorbed into the leaf by FWU: (1) entry into the mesophyll and use for photosynthesis or capacitance, (2) entry into the vasculature, or (3) transpiration back into the atmosphere [7]. This process is important for plant growth and development. At present, the leaves of 233 plant species have been reported to have the ability to absorb water [8]. These plants are widely distributed in tropical mountain cloud forest ecosystems, Mediterranean climate forests and shrub ecosystems, tropical rain forest ecosystems, and desert ecosystems [9–12]. FWU has been found to be a common physiological phenomenon in plants [13–15].

FWU plays an important role in maintaining plant carbon and water balance. Many studies have observed that FWU can be transported to palisade tissues, spongy mesophyll cells, and epidermal cell walls [16, 17], thereby increasing leaf water potential. The increase in water potential will further bring many physiological benefits, such as increasing stomatal conductance, promoting gas exchange, enhancing respiration, and sufficient water also ensures the efficiency of photosynthesis. Lehmann et al. [18] proved that FWU can enter mesophyll cells and participate in photosynthesis. Zhang et al. [19] found that FWU of *Populus euphratica* seedling significantly improved the growth and development ability and chlorophyll fluorescence parameters of its. Some studies have also found that when the leaf surface is wet, it will cause CO_2_ to enter the leaf and inhibit plant photosynthesis to a certain extent. The results of Gerlein-Safdi et al. [20] showed that the presence of dew reduced water loss by 25% in transpiration but also reduced CO_2_ assimilation by 12%. A similar conclusion was drawn by Boanares et al. [21]: FWU has a transient negative effect on CO_2_ assimilation. Therefore, the question of how FWU by other plant affects their water and photosynthetic physiology is worthwhile.

With continuous research development on FWU, it has been found that plants cannot obtain enough water from soil under drought conditions, and the phenomenon of FWU will be more obvious [13]. For example, in arid areas, FWU can significantly increase stomatal conductance (*Gs*) and aboveground biomass of *Bassia dasyphylla* [14], as well as leaf water potential and net photosynthetic rate (*Pn*) of psammophyte [15], thus improving the plant water status [9]. As the slight increase of water potential can also promote the growth of plants [22], FWU plays a decisive role in root growth. The deeper roots are, the more likely it is for plants to get groundwater, thus further promoting plant growth [23]. Therefore, FWU could not only alleviate the negative effects of drought on plants, but also promote plant growth and development [24]. In the past 50 years, global warming has led to changes in the pattern of precipitation [25]. For example, the number of days without rainfall, the change in precipitation characteristics, the increase in drought days in some areas [26], and the fog, dew and precipitation events that cause FWU have changed. Thus, water and carbon balance of plants or ecosystems could experience changes. Therefore, conducting in-depth research on FWU in arid regions and exploring its complex role and potential value will help to evaluate the contribution of FWU to plant communities and its key role in the regional water cycle.

Psammophytes are plants that live in sandy soil based on sand grains, and generally grow in extreme environments such as high temperature, drought, high altitude and so on. It can prevent wind and sand, and protect the ecological environment. For psammophytes living in low water availability soil for a long time [9], leaves absorb condensed water and can significantly improve their own water physiological state, and this strategy is an important water utilization mechanism for them to adapt to harsh water conditions in sandy land [9, 27]. *Calligonum ebinuricum* is a typical psammophyte in arid area with highly developed roots. In order to adapt to extreme drought conditions, its leaves degenerate into assimilating branches, which are the main carbon assimilation organs [28]. However, there are few studies on the water absorption of leaves (assimilating branches) of this type of plants. Therefore, it is more important to further study the FWU of *Calligonum ebinuricum* in arid areas [29, 30]. This is of great significance for the restoration of desert ecosystems and understanding the effects of FWU on carbon-water balance at large scales [8].

At present, the research on FWU is mostly based on short-term experiments, that is, fog-exposure (place the plants in a fog chamber with a humidifier for 6–12 hours) experiments are used to simulate the phenomenon of FWU of plants in high humidity habitats [31]. However, this experiment overlooked two important points. Firstly, in the global ecosystem, leaves are in a wet state for about 120 days every year on average [7], which means that FWU is a long-term process, and short-term fog exposure treatment can not truly reflect the long-term law of FWU of plants in the ecosystem. Secondly, fog exposure simulates the ideal high-humidity habitat, which can well reflect the water absorption potential and ability of plant leaves. However, for plants in arid areas, the high-humidity environment does not exist, and condensed (condensation products formed by water vapor on vegetation and soil surfaces after the surface temperature drops to dew point) water is the main way for FWU [9, 32, 33]. By calculating the local condensation water quantity, it is more reasonable to spray atomized water droplets (distilled water: local condensation water or multiple condensation water) on the leaf surface to simulate condensation water with a spray device (Humidification treatment) [34]. The purpose of this experiment is to explore the physiological response of *Calligonum ebinuricum* under long-term and short-term leaf humidification in arid areas through water potential, gas exchange and fluorescence parameter. Based on previous studies, in most cases FWU improves plant water status and photosynthesis, which in turn promotes plant growth [35], we put forward the following hypotheses: (1) After long-term and short-term FWU, the water potential and photosynthetic rate (*Pn*) of *Calligonum ebinuricum* will increase and water status will be improved; (2) the health status of long-term FWU plants is superior to that of short-term FWU plants.

## Materials and methods

### Study area

Ebinur Lake Wetland National Nature Reserve (82°36’–83°50’E, 44°30’–45°09’N) is located in Jinghe County, Boertala Mongol Autonomous Prefecture, Xinjiang Uygur Autonomous Region, China. This area is located in the lowest-lying land and water salinity concentration center in the western Junggar Basin, with a total area of 2670.85 km^2^. The average annual temperature is 5°C; annual precipitation is 107 mm, and annual evaporation is 1315 mm. The distribution of precipitation during the year is uneven, with higher amounts in summer and lower amounts in winter, and the climate is extremely arid, representing a typical continental arid climate temperature. The typical zonal soils in the study area are gray desert soil, gray palm desert soil, and aeolian sand soil, and the hidden zone soil is salt (salinized) soil, meadow soil, marsh soil, and brown calcareous soil. Under the influence of factors including various soil types, geomorphology, and hydrology, plant communities with rich biodiversity were formed. The main plant community types are xerophytic, super xerophytic, sandy, and halophytic types. The flora of Ebinur Lake Wetland Nature Reserve belongs to the Junggar Desert community in the northern Xinjiang Desert area of the Palaearctic Mongolia New Area. The dominant species are *Kalidium foliatum*, *Poacynum pictum*, *Populus euphratica*, *Horaninowia ulicina*, *Haloxylom ammodendron* and *Calligonum ebinuricum*.

### Sample setting and sample plant selection

**Fig 1:**
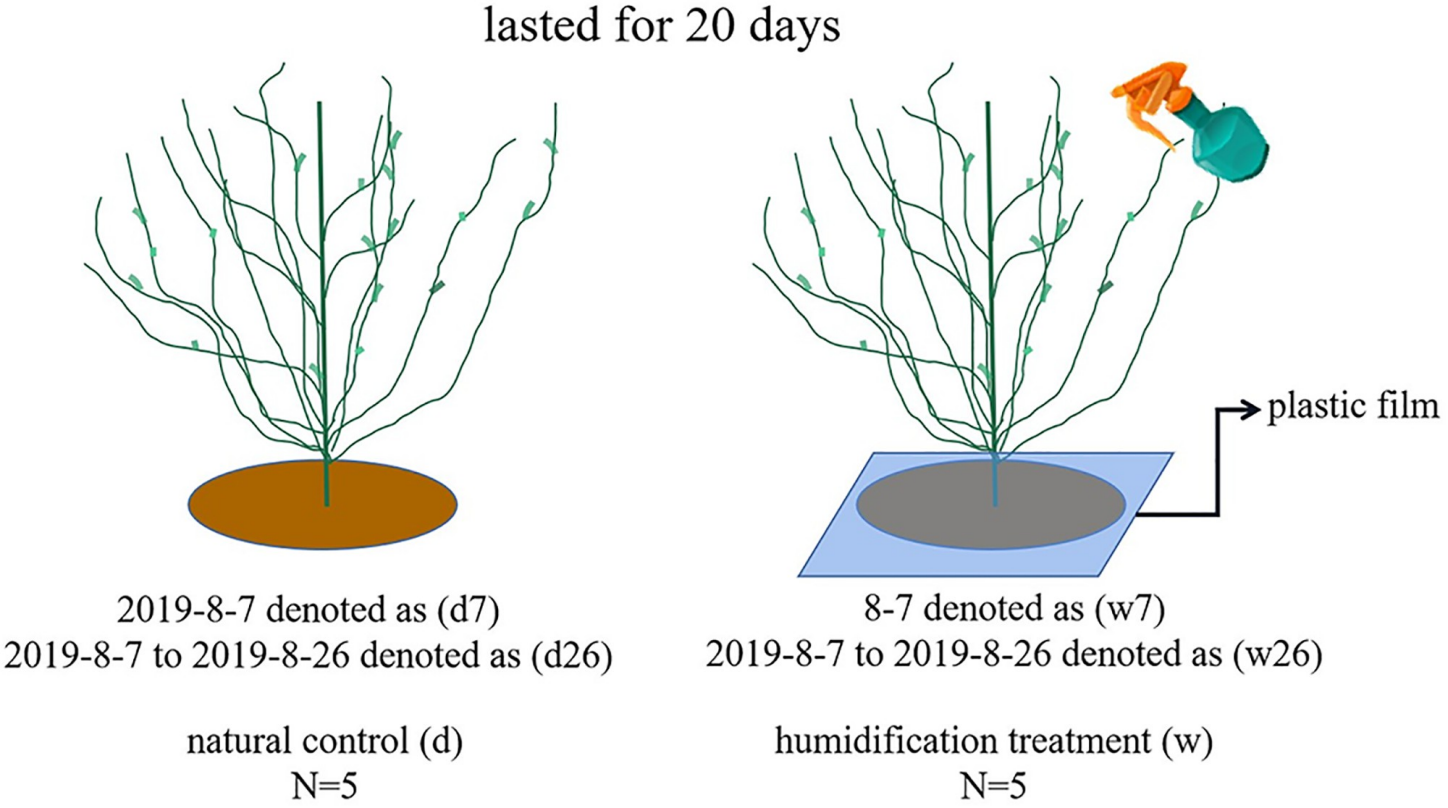
Experimental design. Notes: N = 5: 5 trees of *Calligonum ebinuricum*.

In the Ebinur Wetland National Nature Reserve, which is 6 km to the north of Dongdaqiao Management and Protection Station, 10 healthy and mature *Calligonum ebinuricum* were selected as research objects and were marked. The sampled plant information of *Calligonum ebinuricum* is shown in Table 1. In order to eliminate the errors of the in-situ environment in different plants, the data displayed by the soil temperature and humidity meter and the soil pH (IQ150) meter were used as reference for the selection of experimental plants, and 10 plants with similar growth environment were selected as sample plants. Five trees were randomly selected for leaf humidification treatment and were marked as w (humidification treatment: spraying mist water droplets on the leaf surface by a spraying device filled with distilled water). The remaining five trees without any treatment were marked as d (natural control) and were numbered. Before the experiment (on August 6th), the water potential of the experimental and control groups was measured. There was no significant difference between the two groups (*P* > 0.05; S1 Table), that is, the physiological state of the plant before the experiment was consistent. During the experiment, only the canopies of plants in the humidification treatment were wetted. In order to prevent the water flow on the leaf surface from dropping to the soil surface, polyethylene film was placed under the crown before spraying. Experimental design is shown in Fig 1.

**Table 1:** A questionnaire for the experimental sample of *Calligonum mongolicum*.

Treat	Height/cm	Crown/cm*cm	DBH(BH)/cm
d1	43	85*45	0.4
d2	53	78*73	0.5
d3	30	93*61	0.4
d4	40	70*56	0.4
d5	30	95*80	0.4
w1	35	70*80	0.4
w2	46	85*65	0.4
w3	55	92*65	0.5
w4	47	78*76	0.4
w5	46	110*85	0.5

Notes: Treat includes d and w, d1-d5 means 5 trees of natural control; w1-w5 means 5 trees of humidification treatment. Height is the height of the tree, Crown is the crown width, DBH is the diameter at breast height.

### Field in situ control experiment

From August 7^th^, 2019, to August 26^th^, 2019, the leaves of 5 selected *Calligonum ebinuricum* were humidified at predawn (before sunrise) and evening (after sunset) every day for 20 consecutive days. The measurements on August 7^th^ were recorded as d7 (natural control) and w7 (short-term humidification), and the measurements on August 26^th^ (20 days) were recorded as d26 (natural control) and w26 (long-term humidification).

In this experiment, the average crown width of *Calligonum ebinuricum* was 0.591 ± 0.153 m^2^ (mean ± SD), According to an experiment conducted by Gong [34], the daily condensation per unit area of the plants in the desert area of the Ebinur Basin (0.01 m^2^, 0.31 mm·d^−1^) was obtained, and the amount of water required for humidification was about 183 ml·d^−1^ per plant; 91.5 ml was sprayed at dawn (before sunrise) and evening (after sunset) to simulate the two peaks of water vapor condensation in the canopy under natural conditions. During the experimental period, only one small-scale rainfall event occurred at 19:27:00–20:38:00 on August 17^th^. To avoid the influence of precipitation, all plants were covered with polyethylene film during this period.

### Water potential measurements

After the leaves were humidified on August 7^th^, 2019, water potential plant samples were collected (Each plant selected 2–3 leaves in different directions, i.e. 12 replicates per treatment), and water potential was measured again from 12:00 to 12:30 noon (Beijing time, the same below). After the collection, the experimental sealing film (PM-996, Parafilm, USA) wrapped the gap caused during sampling and brought leaves back to the laboratory quickly. The water potential of the samples was measured with a dew point water potential meter (WP4C, DECAGON, USA) (During the sample test, the leaves were gently wiped with paper towels to remove the influence of surface moisture and dust). The experimental procedure on August 26^th^, 2019, was the same as that on August 7^th^.

### Determination of photosynthetic parameters and chlorophyll fluorescence parameters

A portable photosynthesis system (LI-6400XT, LI-COR, USA) was used to measure photosynthesis of 10 *Calligonum ebinuricum* trees on August 7^th^ and August 26^th^ at noon (12: 00–14: 00; Beijing time), during which time photosynthesis was intense, using a 2 × 3 cm^2^ standard leaf chamber equipped with a LED light source (6400-02B), the reference CO_2_ concentration was set to 400 μmol·mol^-1^, the flow rate was 500 μmol·s^−1^, and the leaf chamber temperature was 30°C.

After the matching value was stable, the measurement was started at 12: 00 and ended at 14:00. The photosynthetically active radiation (PAR) in Ebinur lake was about 1200 mol m^-2^ s^-1^, and atmospheric CO_2_ concentration was about 400 ppm. During the measurement, the leaves were laid flat in the leaf chamber without shielding each other, and the total surface area of the leaves was regarded as the photosynthetic effective area. The measured parameters were the net photosynthetic rate (*Pn*, μmol·m^−2^·s^−1^), stomatal conductance (*Gs*, mol·m^−2^·s^−1^), intercellular carbon dioxide (*Ci*, μmol·m^−2^·s^−1^) and transpiration rate (*Tr*, mmol·m^−2^·s^−1^). Because *Calligonum ebinuricum* leaves are fleshy and scaly, their shape is approximately cylindrical. It is convenient to use vernier calipers (0.05 mm) to measure the leaf diameter and to calculate the total leaf area in the leaf chamber according to the calculation formula of the surface area of a cylinder. The leaf chamber has single-sided lighting, the actual photosynthetic area should be 1/2 of the calculated area [36]. The *Calligonum ebinuricum* leaves to be tested were wrapped in tinfoil, and then the chlorophyll fluorescence parameters (Fluorescence leaf chamber) (6400–40, Li-COR) of the photosynthetic organs were measured after dark treatment for 30 minutes. Before measurement, the instrument parameters were set to the system default values, and the activation light intensity was set to 1200 mol m^-2^ s^-1^. The measurement parameters included the minimum fluorescence (*Fo*), maximum fluorescence (*Fm*), and the maximum photon quantum yield (*Fv/Fm*). Other parameter calculation formulas are as follows [37]:

quantum yield of photosystem II:

$$\Phi_{\text{PS}\;\text{II}}=\left(Fm\prime -Fs\right)/Fm\prime$$ (1)

photochemical quenching:

$$qP=\left(Fm\prime -Fs\right)/\left(Fm\prime -Fo\prime \right)$$ (2)

non-photochemical quenching:

NPQ=Fm/Fm′−1 (3)

electron transfer rate:

$$ETR=PPFD\times \Phi_{\text{PS}\;\text{II}}\times \;0.5\;\times \;0.85$$ (4)

carboxylation efficiency:

CE=Pn/Ci (5)

The photosynthetic and fluorescence parameters were determined for each plant using uniformly growing, healthy and mature leaves, and the measurement was repeated 3 times. Leaves were wiped before measurement to prevent any effects of moisture or dust on the surface of the leaf.

### Statistical analyses

Excel 2013 (Microsoft, USA) was used to sort the data, and two-way repeated measures ANOVA and one-way ANOVA were completed in SPSS 19.0 (IBM Analytics, USA). The effect of experimental treatment (natural control or humidification treatment) on leaf water potential (Ψ_leaf_), photosynthetic gas exchange, and chlorophyll fluorescence was analyzed using two-way repeated measures ANOVA with measurement day/duration (August 7^th^ or August 26^th^) and experimental treatment as predictor variables (experimental treatment×measurement day/duration). If there was an interaction between experimental treatment and measurement day/duration (*P* < 0.05), simple effect was used for further analysis. If there was no interaction (*P* > 0.05), one-way ANOVA was used to further analyze the experimental treatment to determine the influence of humidification treatment and natural control on plant physiological traits. The significance level was uniformly set to α = 0.05. Figures were drawn using Excel 2013 (Microsoft, USA) and Origin 2018 (OriginLab, USA).

## Results

### Changes in water potential in photosynthetic organs of *Calligonum ebinuricum*

**Fig 2:**
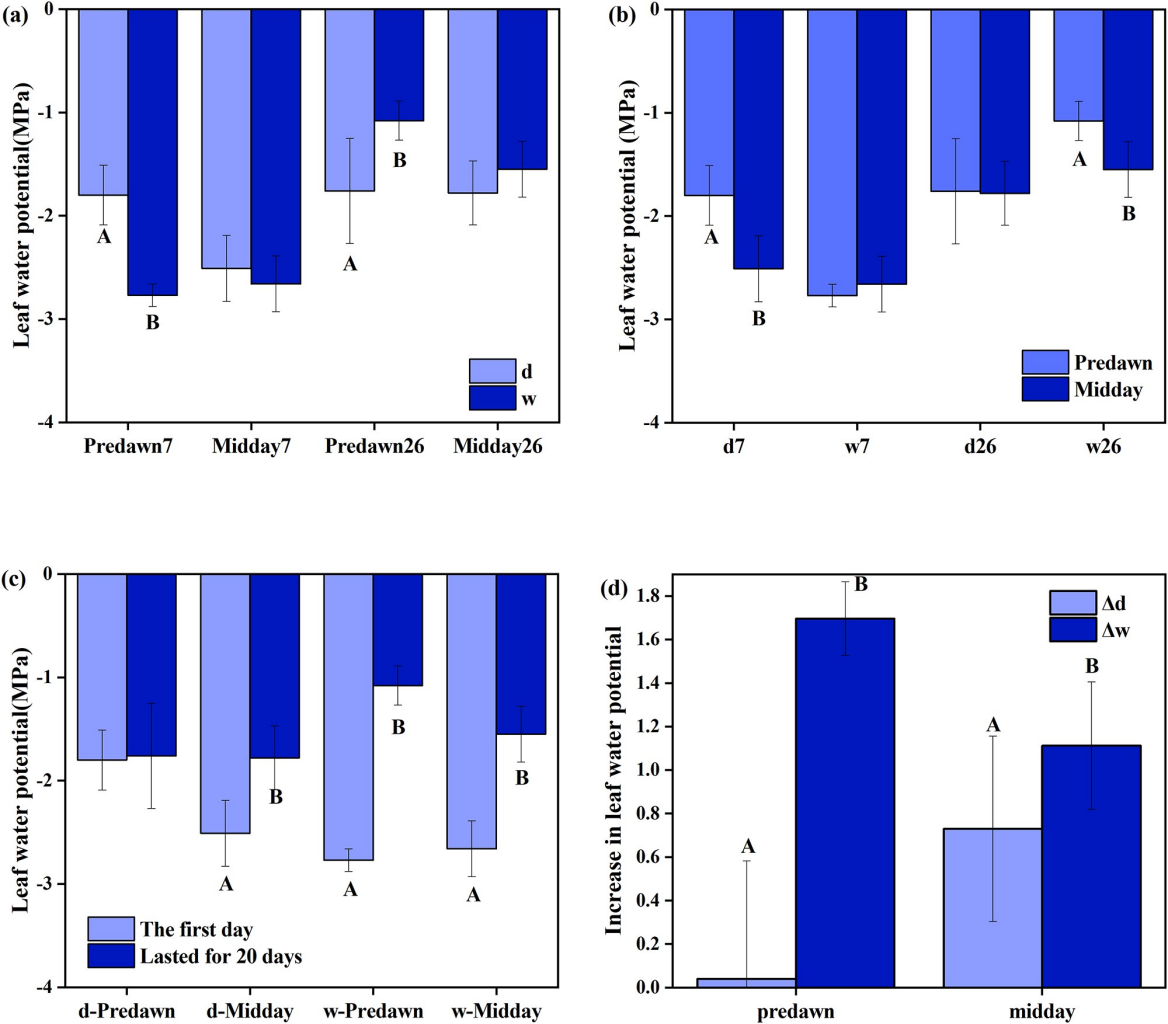
Effect of humidification treatment on the water potential of *Calligonum ebinuricum*. Figure a represents the difference in leaf water potential between the natural control (d) and humidification (w), Predawn 7: leaf water potential of predawn (Ψ_predawn_) on August 7^th^ (i.e. the first day); Midday 7: leaf water potential of midday (Ψ_midday_) on the first day; Predawn 26: Ψ_predawn_ on August 26^th^ (i.e. lasted for 20 days); Midday 26: Ψ_midday_ after 20 days. Figure b represents the difference in Ψ between midday and predawn, d7: natural control on the first day; w7: short-trem humidification; d26: natural control after 20 days; w26: long-term humidification. Figure c represents the difference in Ψ between the first day and 20 days later, d-Predawn: Ψ_predawn_ under natural control; d-Midday: Ψ_midday_ under natural control; w-Predawn: Ψ_predawn_ after humidification; w-Midday: Ψ_midday_ after humidification. Figure d represents the difference in Ψ increment between the first day and continuous experiment 20 days under natural control and humidification, Δd: Ψ difference between the first day and 20 days later; Δw: Ψ difference between the short-term humidification and long-term humidification. Note: Uppercase letters indicate significant differences in leaf water potential. Each bar represents the mean value +SE of 12 repetitions for each treatment.

From Fig 2a, it can be seen that after the humidification treatment, predawn leaf water potential (Ψ_predawn_) was significantly lower than that of the natural control on the first day (Humidification on the first day: short-term humidification) (*P <* 0.05), at noon (Ψ_midday_) the reduction was not significant (*P* > 0.05). After continuous humidification for 20 days (long-term humidification), Ψ_predawn_ was significantly higher than that of the natural control (*P <* 0.05), and Ψ_midday_ increased slightly (*P* = 0.062).

As it can be seen from Fig 2b that on the natural control, Ψ_midday_ was significantly lower than Ψ_predawn_ on the first day (*P <* 0.05), and there was no significant difference between Ψ_midday_ and Ψ_predawn_ after short-term humidification(*P >* 0.05). After 20 days, Ψ did not differ significantly between predawn and noon (*P >* 0.05) under natural control. However, after long-term humidification, Ψ_midday_ was significantly lower than Ψ_predawn_ (*P <* 0.05).

It can be seen from Fig 2c that at predawn, there was no significant difference in Ψ between the 1^st^ and 20^th^ days (*P >* 0.05) under the natural control conditions, and long-term humidification was significantly higher than short-term humidification (*P <* 0.05). However, at noon, the natural control and humidification treatment were significantly higher on the 20^th^ day than the first day (*P <* 0.05).

From Fig 2d, it can be seen that after 20 days of continuous humidification, the water potential increment was significantly higher than that of nature control both at noon and predawn (*P <* 0.05).

### Changes in the gas-exchange of *Calligonum ebinuricum*

**Fig 3:**
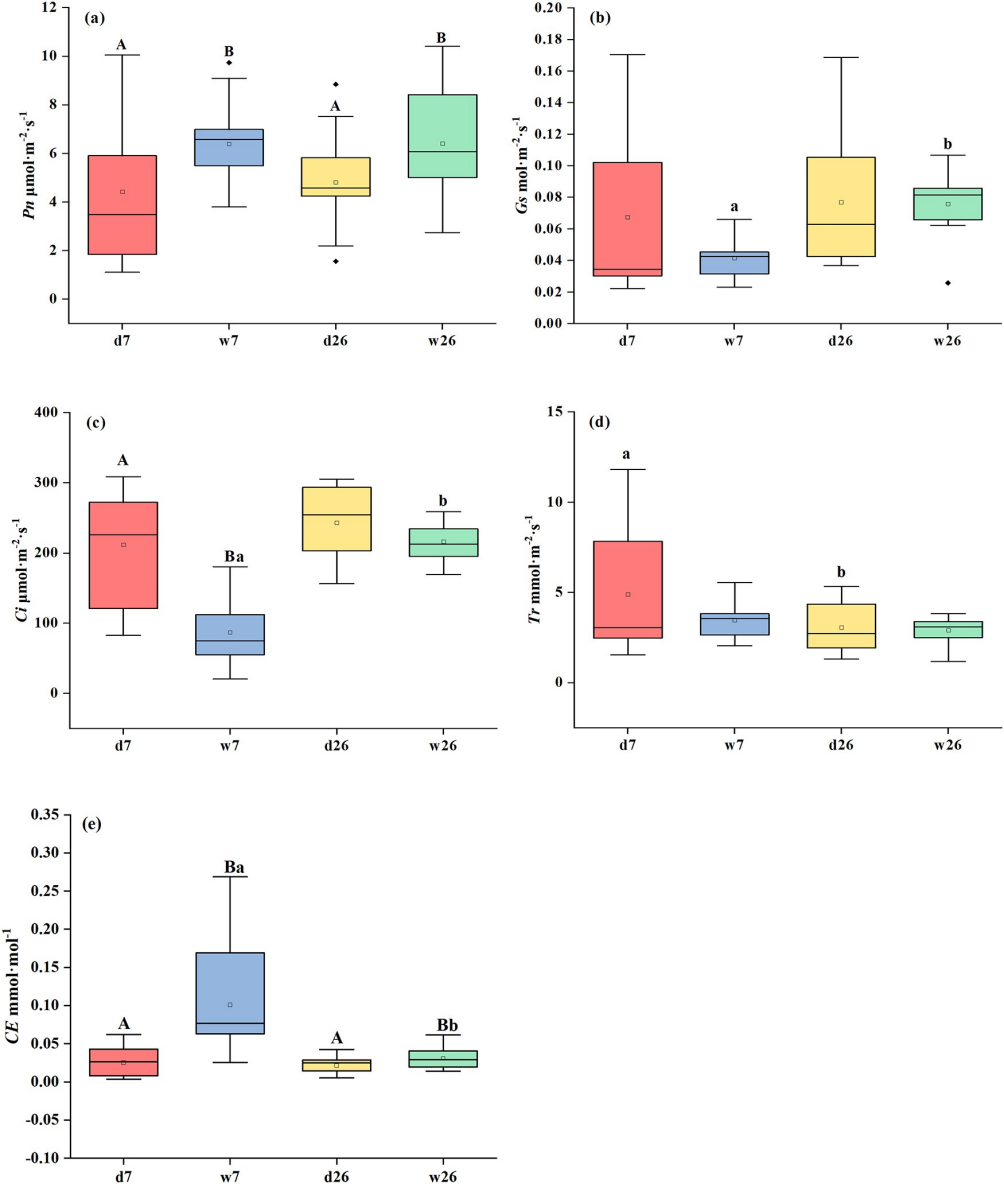
Effects of humidification treatment on the photosynthetic physiology of *Calligonum ebinuricum*. The figure shows the differences between *Pn* (net photosynthetic rate), *Gs* (stomatal conductance), *Ci* (intercellular carbon dioxide), *Tr* (transpiration rate) and *CE* (carboxylation efficiency) under different treatments in the natural control (d7 and d26), short-term humidification (w7) and long-term humidification (w26). Note: Uppercase letters indicate differences between groups (d7 and w7; d26 and w26), lowercase letters indicate differences within groups (d7 and d26; w7 and w26); the absence of a label indicates no difference. Each box includes the mean value +SE of 15 repetitions for each treatment.

The measurements of gas exchange (Fig 3) showed that compared with the natural control (d7), photosynthetic rate (*Pn*) and carboxylation efficiency (*CE*) increased significantly (*P <* 0.05; Fig 3a and 3e) after short-term humidification (w7), while *Ci* decreased significantly (*P <* 0.05; Fig 3c), but *Gs* and *Tr* had no significant changes (*P >* 0.05; Fig 3b and 3d) compared with the natural control (d7).

After long-term humidification (w26),*Pn* and *CE* increased significantly (*P <* 0.05; Fig 3a and 3e), while *Gs*, *Ci* and *Tr* changed little (*P >* 0.05; Fig 3b–3d).

In terms of duration, compared with short-term humidification (w7), *Gs* and *Ci* were significantly increased after long-term humidification (w26) (*P <* 0.05; Fig 3b and 3c), *CE* was significantly decreased (*P <* 0.05; Fig 3e), and *Pn*, *Tr* did not change (*P >* 0.05; Fig 3a and 3d).

### Changes in the chlorophyll fluorescence parameters of *Calligonum ebinuricum*

Compared with the natural control, *Fv/Fm* and *NPQ* increased significantly (*P <* 0.05; Table 2) after short-term humidification (w7), while other parameters changed little (*P >* 0.05; Table 2). After long-term humidification (w26), *Φ*_PS II_, *qP* and *ETR* increased significantly (*P <* 0.05; Table 2), and *Fv/Fm* increased to 0.72 ± 0.03, in the range of 0.7–0.8, the plant reached a healthy state.

**Table 2:** Effects of humidification treatment on the photosynthetic physiology of *Calligonum ebinuricum*.

Fluorescence Parameter	treatment			
	d7	w7	d26	w26
Fo	55.98 ± 33.34	51.20 ± 20.47	54.20 ± 33.66	45.12 ± 16.88
Fm	139.61 ± 93.85	138.66 ± 55.14	204.56 ± 148.39	163.91 ± 58.90
Fv/Fm	0.56 ± 0.12 A	0.63 ± 0.06 Ba	0.51 ± 0.91	0.72 ± 0.03b
Φ_PS II_	0.18 ± 0.05	0.20 ± 0.05	0.19 ± 0.04 A	0.22 ± 0.03 B
qP	0.65 ± 0.15 a	0.73 ± 0.22 a	0.34 ± 0.07 Ab	0.45 ± 0.10 Bb
NPQ	0.21 ± 0.30 Aa	0.61 ± 0.22 B	0.79 ± 0.92 b	0.63 ± 0.18
ETR	139.65 ± 34.79	152.59 ± 38.02	146.68 ± 33.38 A	172.70 ± 23.73 B

Note: d7 and d26 represent the natural controls under August 7^th^ and August 26^th^, respectively; w7 and w26 represent short-term humidification and long-term humidification respectively; Uppercase letters indicate differences between groups (d7 and w7; d26 and w26); and lowercase letters indicate differences within groups (d7 and d26; w7 and w26).

Compared with the short-term humidification (w7), *Fv/Fm* significantly increased (*P <* 0.05; Table 2) and *qP* significantly decreased after long-term humidification (w26) (*P <* 0.05; Table 2).

## Discussion

### Changes in the water potential in photosynthetic organs of *Calligonum ebinuricum*

The water potential is an index reflecting the water deficit of plants [38], and changes in the water potential can reflect the plant water status after FWU [7, 12]. Previous experiments have proved that when leaves are in a high humidity environment or there is water on their surface, they will absorb water through epidermal structures such as stomata, cuticles and trichomes, thus improving the water potential, photosynthesis, and having a positive impact on the survival and growth of plants [27, 39–43].

In this study, after short humidification (w7), the predawn leaf water potential (Ψ_predawn_) of *Calligonum ebinuricum* was significantly lower than that of natural control (*P <* 0.05; Fig 2a). Surprisingly, it is different from the water potential increase in previous studies [12], This phenomenon has not been seen in any existing study of FWU, and when the leaves are instantly humidified, there might present a "stress response". Sudden moisture wetting makes the leaves in a dry state have an instinctive self-protection reaction and reduces stomatal conductance (Fig 3b) to prevent the excessive invasion of water, which leads Ψ_leaf_ decrease. Finaliy Ψ_leaf_ was recovered at midday (Fig 2a and 2b). Therefore, in the early stage of leaf humidification (short-term FWU), the water condition of *Calligonum ebinuricum* was not improved.

After long-term humidification (w26), Ψ_leaf_ was significantly increased compared with the natural control. In other words, during the long-term humidification process, the FWU phenomenon was generated, and the water condition was obviously improved. which was consistent with the results of previous studies [7].

Ψ_leaf_ under long-term humidification was significantly higher than short-term humidification (w7) no matter predawn or midday (*P* < 0.05; Fig 2c). The leaf water potential decreased after short-term humidification, When the plant leaves continued to humidify, FWU water potential driving gradient reversal increased, the FWU ability increased, and the plant water status improved, this indicates that long-term FWU has the potential to maintain high water potential of plants, the improvement in plant water status can translate directly to enhanced photosynthesis (Fig 3a). This is consistent with the results of other scholars [7, 18, 44–48]. However, in the natural control, midday leaf water potential (Ψ_midday_) after 20 days was also significantly higher than that on the first day (*P* < 0.05; Fig 2c). This result makes it impossible to fully determine whether the Ψ_leaf_ under long-term humidification treatment is significantly higher than that under short-term humidification treatment is due to humidification treatment. The two-way repeated measures ANOVA, it was found that the interaction between the Measurement day/duration and the experimental treatment (natural control and humidification treatment) had a certain effect on leaf water potential (Ψ_predawn_: F = 54.288, *P* < 0.0001; S2 Table) (Ψ_midday_: F = 3.353, *P* = 0.078; S2 Table). We also use the Ψ_leaf_ increment for further determination. After continuous humidification, the increase of water potential was significantly higher than that under natural control (*P* < 0.05; Fig 2d), which further indicates that long-term humidification has the potential to increase the water potential. In the long run, FWU as a supplementary water source can improve the water status of *Calligonum ebinuricum*.

### Gas-exchange and fluorescence physiological response of *Calligonum ebinuricum*

Gas exchange parameters reflect the photosynthetic capacity of plants. Chlorophyll fluorescence parameters reflect the ability of plant leaves to absorb, deliver, dissipate and distribute light energy [44]. Compared with gas exchange parameters, chlorophyll fluorescence parameters are more suitable for further analysis of non-stomatal limiting factors of photosynthesis.

In this research, after short-term (w7) humidification, The stomatal conductance and transpiration rate of *Calligonum ebinuricum* decreased somewhat, but the magnitude was not significant, which was similar to the conclusion of Boanares’ [21]. It may be that leaf wetting changed the energy balance of the leaves, lowering leaf temperature, and causing the stomata to close, and there will be a phenomenon of transpiration inhibition. Transpiration inhibition supplemented plant water, result the gap between Ψ_midday_ and Ψ_predawn_ was significantly reduced (Fig 2b).

And after short-term humidification, the net photosynthetic rate (*Pn*) of *Calligonum ebinuricum* was significantly increased (*P <* 0.05; Fig 3a). the change trend of intercellular CO_2_ concentration (*Ci*) was inconsistent with *Pn* (Fig 3). The increase of photosynthesis was mainly regulated by non-stomatal factors [45]. In addition, stomatal does not work independently, but has certain correlation with other parameters [46]. In nature, plants will control water absorption by photosynthetic organs by opening and closing stomata. Stomatal conductance directly affects intercellular CO_2_ concentration, and it is also the main factor of transpiration rate. In this experiment, the change trends of intercellular CO_2_ concentration and transpiration rate were consistent with stomatal conductance (Fig 3). However, after short-term humidification, intercellular CO_2_ concentration was significantly different from that of natural control (*P <* 0.05; Fig 3c), while stomatal conductance and transpiration rate was not significantly different (*P >* 0.05; Fig 3c and 3d). The reason for this is that the increase in photosynthetic rate being the result of the increase in carboxylation activity (Fig 3e) in the leaves (which is not regulated by stomatal factors), resulting in the decrease in intercellular CO_2_ concentration [46], which led to significant difference. By analyzing chlorophyll fluorescence parameters and water potential value, it was found that the water potential decreased significantly (*P* < 0.05; Fig 2a), the maximum photochemical efficiency of PS II (*Fv/Fm*) and the non-actinic fluorescence quenching coefficient (*NPQ*) increased significantly (*P*<0.05; Table 2), but *Fv/Fm* did not reach 0.7, indicating that the decrease of water potential directly affects the light reaction process and leads to photoinhibition. *Calligonum ebinuricum* protects itself by increasing heat dissipation to resist photoinhibition [47]. We conclude that after short-term humidification, there is no positive effect on the photoreaction phase. The drought stress state of plants did not really improve.

After long-term humidification (w26), the stomatal conductance was significantly higher than that of the short-term humidification (*P <* 0.05), but only slightly lower than that of the natural control (Fig 3b). This phenomenon is considered as an adaptive strategy for plants to cope with long-term humidification. The *Pn* of *Calligonum ebinuricum* was also significantly increased (*P <* 0.05; Fig 3a). The increase of photosynthesis was also mainly regulated by non-stomatal factors [45]. The actual light energy conversion efficiency (*Φ*_PS II_), opening degree (*qP*) and electron transport rate (*ETR*) were significantly higher (*P <* 0.05; Table 2) [48], the maximum photochemical efficiency of PS II (*Fv/Fm*) increased from 0.51 ± 0.91 to 0.72 ± 0.03, within the range of 0.7–0.8, indicating that continuous humidification could alleviate drought stress and make plants in a healthy state [46]. This is consistent with the results of previous studies, such as, Zhang *et al*. [19] showed that the chlorophyll fluorescence parameters (*Φ*_PS II_, *ETR*) of *Populus euphratica* seedlings could be improved after FWU. Combining water potential, gas exchange parameters as well as chlorophyll fluorescence parameters, it was found that after long-term FWU the improvement of water status of plants directly affected the light reaction stage, such as water-splitting reaction, electron transfer, and photophosphorylation. Firstly, the increase of water potential in plant leaves promotes the water-splitting reaction, and then the increase of electron transfer efficiency (ETR) and the opening degree (*qP*) of the PS II reaction center enhances the ability of electron transfer and photochemical reaction by using captured photon energy [49, 50]. Therefore, the H^+^ generated in the water-splitting reaction can be transferred more efficiently, one part of which participates in the photophosphorylation process for the synthesis of intermediate products NADPH and ATP, and the other part is used for the synthesis of sugars in the carbon reaction process [51]. Among them, the formation of intermediate products further promotes the occurrence of carbon reaction, and the carboxylation efficiency is significantly enhanced [52] (*P* < 0.05; Fig 3e), therefore, the net photosynthetic rate (*Pn*) increased significantly (*P* < 0.05; Fig 3a). This is consistent with the results of previous studies, FWU increases leaf water potential, the increase in water potential further promotes gas exchange, and enhances respiration. Sufficient water also ensures the efficiency of photosynthesis [12, 23, 35]

Comparing long-term and short-term humidification, we found that leaf water potential, stomatal conductance, intercellular CO_2_ concentration, *Fv/Fm*, *qP*, were significantly increased, further proving that long-term FWU can improve plant health and photosynthesis.

## Conclusions

On the whole, after short-term FWU, the water condition and health condition of plants have not improved, which is contrary to the hypothesis presented here. While after long-term FWU, *Calligonum ebinuricum* leaves will reduce direct light and total solar radiation, prevent light inhibition of photosynthesis, improve the chlorophyll fluorescence activity of leaves, alleviate a large amount of water loss caused by transpiration, promote the CO_2_ fixation ability of leaves, improve the light energy utilization efficiency, and ultimately enhance photosynthesis [53]. This is in agreement with the research results of other scholars [7, 40]. Long-term FWU can alleviate the negative effects of drought on plants to some extent, enabling plants to perform normal physiological activities [24]. Due to the small increase in water potential can also promote plant growth, so long-term FWU plays a decisive role in root growth, and the deeper the root system, the more likely the plant is to obtain groundwater, thereby further promoting plant growth [23], such a virtuous cycle has further amplified the benefits of FWU in plant survival, growth and ecological adaptation.

## Supporting information

S1 Table Water physiological indexes of *Calligonum ebinuricum* before experimental treatment (August 6^th^). (DOCX)

S2 Table Two-way repeated measurement ANOVA analysis table of leaf water potential, photosynthetic parameters and chlorophyll fluorescence parameters. (DOCX)
