# Supplementary material for: Response of water and photosynthetic physiological characteristics to leaf humidification in Calligonum ebinuricum
Source: PLoS One. 2023 May 4;18(5):e0285130. doi: 10.1371/journal.pone.0285130 (PMC10159122; doi:10.1371/journal.pone.0285130)
Supplement: S1 Table — (DOCX) [file pone.0285130.s001.docx]

|  | d(n=5) | w(n=5) | *P-value* |
| --- | --- | --- | --- |
| Ψpredawn | -2.05 ± 0.03 | -2.21 ± 0.14 | 0.312 |
| Ψmidday | -2.65 ± 0.15 | -2.51 ± 0.09 | 0.462 |

Table S1 Water physiological indexes of *Calligonum ebinuricum* before experimental treatment (August 6th)

Note: *P* < 0.05
