# Supplementary material for: Response of water and photosynthetic physiological characteristics to leaf humidification in Calligonum ebinuricum
Source: PLoS One. 2023 May 4;18(5):e0285130. doi: 10.1371/journal.pone.0285130 (PMC10159122; doi:10.1371/journal.pone.0285130)
Supplement: S2 Table — (DOCX) [file pone.0285130.s002.docx]

Table S2: Two-way repeated measurement ANOVA analysis table of leaf water potential, photosynthetic parameters and chlorophyll fluorescence parameters.

| Response variable | Predictor variable | *F* | *P* | Response variable | Predictor variable | *F* | *P* |
| --- | --- | --- | --- | --- | --- | --- | --- |
| Ψ_predawn_ | Measurement day | 83.676 | **<0.0001** | Fo | Measurement day | 0.521 | 0.476 |
|  | treatment | 13.325 | **0.001** |  | treatment | 0.921 | 0.344 |
|  | Measurement day×treatment | 54.288 | **<0.0001** |  | Measurement day×treatment | 0.156 | 0.695 |
| Ψ_midday_ | Measurement day | 128.318 | **<0.0001** | Fm | Measurement day | 4.802 | **0.035** |
|  | treatment | 1.394 | 0.248 |  | treatment | 0.705 | 0.407 |
|  | Measurement day×treatment | 3.353 | 0.078 |  | Measurement day×treatment | 0.930 | 0.342 |
| Pn | Measurement day | 0.164 | 0.671 | Fv/Fm | Measurement day | 0.051 | 0.822 |
|  | Treatment | 6.908 | **0.014** |  | treatment | 1.701 | 0.201 |
|  | Measurement day×Treatment | 0.185 | 0.688 |  | Measurement day×treatment | 0.452 | 0.506 |
| **Gs** | Measurement day | 18.396 | **<0.0001** | *Φ*_PS II_ | Measurement day | 1.183 | 0.287 |
|  | Treatment | 1.365 | 0.253 |  | treatment | 5.425 | **0.028** |
|  | Measurement day×Treatment | 5.850 | **0.022** |  | Measurement day×treatment | 0.461 | 0.503 |
| **Ci** | Measurement day | 46.497 | **<0.0001** | qP | Measurement day | 68.382 | **<0.0001** |
|  | Treatment | 25.713 | **<0.0001** |  | treatment | 5.200 | **0.031** |
|  | Measurement day×Treatment | 17.268 | **<0.0001** |  | Measurement day×treatment | 0.158 | 0.695 |
| **Tr** | Measurement day | 12.736 | **0.001** | NPQ | Measurement day | 5.872 | **0.023** |
|  | Treatment | 1.770 | 0.194 |  | treatment | 0.658 | 0.425 |
|  | Measurement day×Treatment | 3.535 | 0.071 |  | Measurement day×treatment | 5.139 | **0.032** |
|  |  |  |  | ETR | Measurement day | 2.090 | 0.160 |
|  |  |  |  |  | treatment | 5.694 | **0.025** |
|  |  |  |  |  | Measurement day×treatment | 0.486 | 0.492 |

Note: Treatment: natural contral and humidification treatment; Measurement day/duration: August 7^th^ (the first day) and August 26^th^ (lasting for 20 days); Ψ_predawn_: predawn leaf water potentials; Ψ_midday_: midday leaf water potential; *Pn*: net photosynthetic rate; *Gs*: stomatal conductance; *Ci*: intercellular carbon dioxide; *Tr*: transpiration rate; *Fo*: minimum fluorescence; *Fm*: maximum fluorescence; *Fv/Fm*: maximum photon quantum yield; *Φ*_PS II_: quantum yield of photosystem II; *qP*: photochemical quenching; *NPQ*: non-photochemical quenching; *ETR*: electron transfer rate.
